# Associations between human milk oligosaccharides and infant growth in a Bangladeshi mother–infant cohort

**DOI:** 10.1038/s41390-023-02927-1

**Published:** 2023-12-05

**Authors:** Miranda G. Loutet, Arash Narimani, Huma Qamar, Chloe Yonemitsu, Lisa G. Pell, Abdullah Al Mahmud, Tahmeed Ahmed, Lars Bode, Diego G. Bassani, Daniel E. Roth

**Affiliations:** 1 The Hospital for Sick Children, Toronto, ON M5G 0A4 Canada; 2 The University of Toronto, Toronto, ON M5S 1A1 Canada; 3 University of California San Diego, San Diego, CA USA; 4 Nutrition and Clinical Services Division, icddr,b, Dhaka, Bangladesh

## Abstract

**Background:**

We aimed to estimate associations between human milk oligosaccharides (HMOs) and infant growth (length-for-age (LAZ) and weight-for-length (WLZ) *z*-scores) at 12 months postnatal age.

**Methods:**

In this secondary analysis of data from a maternal vitamin D trial in Dhaka, Bangladesh (*N* = 192), absolute concentrations of HMOs were measured in 13 ± 1 week(s) postpartum milk samples, infant anthropometric measurements were obtained soon after birth and at 12 months postpartum, and infant feeding was classified during 6 months postpartum. Associations between individual HMOs or HMO groups and LAZ or WLZ were estimated by multivariable linear regression adjusting for infant feeding pattern, maternal secretor status, and other potential confounders.

**Results:**

The concentrations of 6’sialyllactose, lacto-*N*-neotetraose, and the non-fucosylated non-sialylated HMOs were inversely associated with LAZ at 12 months of age, whereas the fucosylated non-sialylated HMO concentration was positively associated with LAZ at 12 months. These associations were robust in analyses restricted to infants who were primarily exclusively/predominantly fed human milk during the first 3 (or 6) months.

**Conclusions:**

Since HMOs are both positively and negatively associated with postnatal growth, there is a need for randomized trials to estimate the causal benefits and risks of exogenously administered HMOs on infant growth and other health outcomes.

**Impact:**

- 6’sialyllactose, lacto-*N*-neotetraose, and the non-fucosylated non-sialylated human milk oligosaccharides (HMOs) were inversely associated with length-for-age *z*-scores (LAZ) at 12 months, whereas the fucosylated non-sialylated HMO concentration was positively associated with LAZ at 12 months among Bangladeshi infants.
- Associations between individual and grouped HMOs with infant length growth at 12 months were as strong or stronger in analyses restricted to infants who were exclusively or predominantly fed human milk up to 3 (or 6) months.
- Randomized trials are needed to characterize the effects of specific HMOs on infant growth, particularly in countries where postnatal linear growth faltering is common.

## Introduction

Human milk is a complex biological fluid with numerous interacting components that are related to infant health outcomes^1^ and which promote healthy infant growth.^2^ Human milk oligosaccharides (HMOs) are complex sugars that comprise the third most abundant component of breast milk. Approximately 200 different HMO structures have been identified to date.^3,4^ HMOs are minimally digested by the infant, so most reach the colon intact^5,6^ where they are metabolized by commensal bacteria, resulting in metabolites such as short chain fatty acids that may exert local effects on the host intestinal mucosa.^4,7^ HMOs can also serve as soluble decoy receptors that block the binding of pathogens to epithelial cells.^3,8,9^ A very small fraction of HMOs are absorbed, entering the infants systemic circulation and likely reaching other organs such as the liver, brain, respiratory and urinary tract.^10–13^ Between-individual variation in the amount and composition of HMOs is primarily driven by differential expression of galactoside α1-2-fucoslyltransferase (FUT2) and galactoside α1-3/4-fucoslyltransferase (FUT3) responsible for adding fucose to the HMO backbone in various linkages (i.e., fucosylation), corresponding to Secretor and Lewis blood group status, respectively;^14,15^ sialyltransferases further involved in adding sialic acid to the HMO backbone in different linkages to either the terminal galactose or the internal *N*-acetylglucosamine.^10^ Individual HMOs can be classified based on fucosylation and sialylation and further subclassified by the specific linkages involved. Factors such as maternal BMI, maternal age, parity, maternal diet, mode of delivery, infant gestational age, infant sex, and lactation stage are also associated with HMO concentrations.^16–24^

HMOs are believed to affect infant growth via prebiotic effects that influence the composition of the infant gut microbiota. Animal models have shown that supplementation with purified sialylated bovine milk oligosaccharides produced a microbiota-dependent change in bone morphology.^25,26^ Observational studies^27–35^ and small randomized control trials with HMO-supplemented formula^36–38^ have shown associations between specific HMOs and infant linear growth. Early-infant growth is an indicator of the risk of morbidity and mortality and is associated with long-term functional and social outcomes in low- and middle-income countries.^39–46^ Lacto-*N*-neotetraose (LNnT), an HMO that has been added as a prebiotic to some infant formulas, was observed to be inversely related with infant linear growth in several observational studies.^29,30,32,33,35^ However, findings for other specific HMOs have been inconsistent among different study populations. While human milk feeding practices (e.g., duration of exclusive human milk feeding, volume of human milk consumed) and their underlying influences (e.g., socioeconomic status) may confound HMO–growth associations, factors that relate to the volume of breast milk consumed also directly affect the extent to which an infant is exposed to HMOs and thereby are also expected to modify HMO–growth associations.^32^ In this study, we aimed to estimate the associations between exposure to individual HMOs and groups of structurally similar HMOs at 3 months of age and infant growth at 12 months of age, accounting for infant feeding patterns, in a cohort of mother–infant pairs in Dhaka, Bangladesh.

## Methods

### Study design and population

This was a secondary analysis of data collected in the Maternal Vitamin D for Infant Growth (MDIG) trial, for which methods were previously described.^47^ In brief, 1300 women were enrolled in their second trimester of pregnancy (17–24 weeks gestation) and randomized to one of five treatment arms: 0 IU/week (placebo), 4200 IU/week, 16,800 IU/week, or 28,000 IU/week vitamin D3 from enrolment to birth followed by placebo until 6 months postpartum, or 28,000 IU/week vitamin D3 from enrolment to 6 months postpartum. Participants were enrolled at the Maternal Child Health Training Institute (MCHTI) in Dhaka, Bangladesh in 2014–2015 and followed prospectively in the community to 2 years postpartum at scheduled study visits. The MDIG trial received ethics approval from the Research Ethics Board (REB) at The Hospital for Sick Children (#1000039072) and the Institutional Review Board at the International Centre for Diarrhoeal Disease Research, Bangladesh (icddr,b) (PR-13055). Secondary uses of data and samples for HMO-related sub-studies were also separately approved by the REB at The Hospital for Sick Children (#1000061001).

Individuals were eligible to be included in this HMO sub-study if they were randomized to receive either placebo or 28,000 IU/week of vitamin D3 in the prenatal and postpartum period (28,000/28,000 IU/week vitamin D3 group), had a human milk sample collected at approximately 3 months postpartum, had maternal height and weight recorded at approximately 12 months postpartum, and had infant length and weight recorded at approximately 12 months postpartum. Among those who were eligible, a simple randomization scheme was applied to select 96 participants from each of the placebo and vitamin D treatment groups for a total sample size of 192.^48^ The placebo and highest dose of vitamin D study arms were chosen to test whether vitamin D supplementation had an effect on HMO composition; however, as previously reported,^48^ vitamin D had no effect, so the groups were combined in all analyses presented here, and supplementation group was included as a covariate in multivariable models.

### Data and biological sample collection

Details of human milk sample collection and laboratory methods to measure the absolute concentrations of 19 HMOs in this study population were previously described.^48^ In brief, participants hand-expressed mid-feed human milk samples at approximately 3 months ± 1 week postpartum. The samples were frozen in 1.5 ml aliquots at approximately −70 °C until they were transferred to The Hospital for Sick Children on dry ice for long-term storage at −80°C. A 100 µl aliquot of each human milk sample included in this HMO sub-study was analyzed at the University of California, San Diego (UCSD).

Pregnant individuals were enrolled at MCHTI between 17 and 24 weeks gestation and followed-up with their infants to 12 months postpartum. Anthropometric measurements used in this sub-study (weight, length) were those collected at the earliest age within the first 45 days of life (referred hereinafter as early-infant size) and at 12-months postpartum visits (364–394 days of age) (referred hereinafter as attained size). In MDIG, length (crown to heel in the supine position) and weight measurements were measured according to procedures adapted from standardized operating procedures from the Intergrowth-21st study.^49^ Two study personnel independently measured each infant and the paired measurements were compared against a threshold difference (7 mm for length and 50 g for weight) to determine if repeat measurements were required.^47^ Maternal age, gravidity, maternal education, and data used to derive the household asset index quintile were self-reported during the prenatal baseline visit. Mode of delivery and infant sex were collected at birth. Gestational age was based on recall of last menstrual period (LMP) and/or gestational ultrasound at the enrolment visit and date of birth. Weekly postnatal routine visits continued to 6 months postpartum, during which study personnel collected data on infant feeding practices, health/vital status and medical events.

### Variables

Oligosaccharide analysis was conducted using high-performance liquid chromatography and absolute concentrations of 19 HMOs (in nmol/ml) were quantified as previously described.^18,50–54^ Mothers were defined as *secretors* if their human milk sample had a 2’-fucosyllactose (2’FL) concentration greater than 350 nmol/ml. HMOs were categorized into five groups based on shared chemical characteristics, which had been previously defined^29^ and used in analyses in the same study population.^48^

Length-for-age (LAZ) and weight-for-length (WLZ) *z*-scores were estimated based on raw lengths and weights. WLZ was estimated using the WHO growth standards.^55^ For all birth length measurements (within 48 h of birth) LAZ was derived using the Intergrowth-21st^56^ newborn standards to standardize for sex and gestational age at birth. For length measurements thereafter, Intergrowth-21st postnatal standards^57^ were used to derive gestational-age-standardized LAZ for preterm infants who were less than 64 weeks of postmenstrual age and WHO growth standards were used for all other length measurements. LAZ at 12 months was the primary outcome of this study and WLZ at 12 months was the secondary outcome, both referred to as attained size in statistical models. Infant feeding pattern was derived for each week of data based on WHO definitions^58,59^ and dichotomized into two categories: exclusive/predominately fed with human milk (exclusive: only consumed human milk; predominant: consumed human milk and other liquids excluding non-human milk and formula) or partially/not fed with human milk (partial: consumed human milk and any food or liquid including non-human milk and formula). Missing infant feeding data were imputed based on the most recent preceding week with observations up to the midpoint of the period of missing data and based on the closest subsequent week in which feeding status was observed after the midpoint of the period of missing data. If there was an odd number of weeks in which infant feeding data were missing, data from the week after the period of missingness were used for the extra week. For example, if there were 5 consecutive weeks with missing infant feeding data, the first two weeks were imputed based on the prior week and the last three weeks were imputed based on the week after the period of missingness. If there was a period with missing infant feeding data starting at birth, the entire period of missing feeding data was imputed based on the first known week of data after the period of missingness. The infant feeding pattern during the first 3 and 6 months postpartum was summarized as the infant feeding pattern that occurred for the majority of weeks up to 3 and 6 months (including weeks with imputed data on feeding pattern), respectively. For example, if an infant was exclusive/predominately fed with human milk each week for more than six weeks in the first 3 months of life, they were categorized as primarily exclusive/predominately fed with human milk during the first 3 months postpartum. Additionally, if an infant was exclusive/predominately fed with human milk each week for more than 13 weeks in the first 6 months of life, they were categorized as primarily exclusive/predominately fed with human milk during the first 6 months of life. A continuous infant feeding variable was derived using the total number of weeks for which an infant was exclusive/predominantly fed with human milk during the first 3 months postpartum.

### Statistical analysis

Baseline characteristics of the study population were described using mean and standard deviation (SD) or median and interquartile range (IQR) for continuous variables and the number and proportion for categorical variables. Absolute concentrations were rescaled to 100 nmol/ml for regression modeling.

To estimate the association between each individual HMO and attained infant size, multivariable-adjusted linear regression was conducted with LAZ (or WLZ) at 12 months regressed on each individual HMO and the following covariates: gravidity, household wealth, maternal body mass index (BMI) at 12 months postpartum (the best available measure of maternal habitual BMI), maternal age, mode of delivery, gestational age, infant sex, vitamin D supplementation group, maternal secretor status, and infant feeding status up to 3 months (up to the time of collection of the milk samples in which HMO concentrations were measured).

If there was evidence of an association using the model for attained size, the multivariable-adjusted linear regression was conducted again with the same set of covariates and early-infant LAZ (or WLZ), which are referred to the baseline-adjusted models. Silverberg et al. conducted growth analyses of the same study population using multiple modeling approaches including the baseline-adjusted ANCOVA model and a conditional model with residuals, which yielded similar estimates.^60^ Therefore, the baseline-adjusted method was selected due to its more straightforward interpretation compared to the other models. To mitigate false-positive associations due to multiple testing, we applied the Bonferroni–Holm method to adjust the significance level whenever one individual HMO or one structurally similar group of HMOs was the primary exposure. We set a less stringent target alpha of 0.10 to assess evidence of an association in attained-size models and as a threshold to proceed with the baseline-adjusted model. In the final baseline-adjusted models, we set a target alpha of 0.05 to determine significance. This gate-keeper modeling approach between attained-size and baseline-adjusted models was done to further reduce the chance of false-positive findings from multiple testing. From the separate baseline-adjusted models, if HMOs were significantly associated with LAZ (or WLZ) they were further included together in a final model to assess if the effect estimates attenuated due to correlations among the HMOs that were associated with the outcomes. Pearson correlation coefficients between the significant individual HMOs and stratified by secretor status were also reported to assess correlations among the HMOs by secretor status and appropriateness of including secretor status as a confounder to adjust for variation between HMOs.

Several secondary analyses were conducted. First, to assess effect estimates among the subset of infants who received the greatest amount of human milk during the first 3 (or 6) months postpartum, the baseline-adjusted models that were significant among all infants were also undertaken restricted to infants who primarily exclusively/predominantly fed with human milk during the first 3 (or 6) months postpartum. Although effect modification analyses were not possible due to the small sample size in groups stratified by infant feeding pattern, baseline-adjusted models were also restricted to infants who primarily partially/did not feed with human milk for the majority of the time during the first 3 (or 6) months postpartum. Furthermore, the same modeling strategy was conducted for each of the five HMO groups based on structure, total concentration of HMOs (sum of all 19 HMOs), and using maternal secretor status as a binary exposure rather than the individual or grouped HMO concentrations.

Sensitivity analyses were conducted by changing the way certain covariates were derived in order to assess whether inferences from the models remained the same. The same modeling strategy was conducted for four alternative HMO groups based on specific linkages. Additionally, the same modeling strategy was conducted adjusting for the continuous infant feeding covariate of the total number of weeks exclusive/predominantly fed human milk during the first 3 months postpartum instead of the binary infant feeding variable. Baseline-adjusted models were also conducted restricted to infants with baseline LAZ measurements within 2 days of birth and using the earliest possible LAZ measurement without any limit placed on the age as a complete case analysis without any missing baseline LAZ measurements. Given that the range of HMO concentrations varied substantially by HMO,^48^ standardizing all HMOs to the same absolute scale may affect the interpretation about the relative magnitude of effect, so significant baseline-adjusted models were also constructed for HMOs standardized to their respective IQR. For HMOs/HMO groups for which primary findings were significant, a post hoc sensitivity analysis was conducted in which the sample was restricted to term infants. Statistical analyses were conducted using STATA 17.0.

## Results

Characteristics of the study population are reported in Table 1. There were 14 infants with a missing early-infant LAZ measurement (i.e., within 45 days of life) but there were no significant differences in infant or maternal characteristics between those who had a early-infant LAZ measurement and those who did not (Supplementary Table 1). Infant feeding data was imputed for 9.0% (224/2496) of visits up to 3 months postpartum and 9.5% (476/4992) of visits up to 6 months postpartum.

**Table 1:** Characteristics of mother–infant pairs included in the HMO sub-studies of the MDIG trial.

Characteristic	*n* (%) or median (25th, 75th percentile), or mean (SD)
*Total, n*	192
*Maternal characteristics*	
Mother age at enrolment in years, median (IQR)	23 (20, 27)
Gravidity, median (IQR)	2 (1, 3)
Vitamin D treatment allocation, *n* (%)	
Placebo	96 (50)
28,000/28,000 IU/week	96 (50)
Postpartum maternal BMI, mean (SD)	24 (4.2)
Maternal education, *n* (%)	
No education	6 (3.1)
Primary incomplete	48 (25)
Primary complete	25 (13)
Secondary incomplete	74 (38)
Secondary complete or higher	39 (20)
Household asset index quintile^a^, *n* (%)	
1	32 (17)
2	34 (18)
3	50 (26)
4	33 (17)
5	43 (22)
Secretor status, *n* (%)	
Secretor	127 (66)
Non-secretor	65 (34)
*Infant characteristics*	
Gestational age categorization, *n* (%)	
Preterm (<37 weeks)	14 (7.3)
Term (≥37 weeks)	178 (93)
Mode of delivery, *n* (%)	
Vaginal	94 (49)
Cesarean section	98 (51)
Infant sex, *n* (%)	
Male	95 (49)
Female	97 (51)
Infant feeding pattern during the first 3 months postnatally, *n* (%)	
Primarily partially/not fed human milk^b^	24 (12)
Primarily exclusively/predominantly fed human milk	168 (88)
Infant feeding pattern during the first 6 months postnatally, *n* (%)	
Primarily partially/not fed human milk^b^	45 (23)
Primarily exclusively/predominantly fed human milk	147 (76)
Early-infant length-for-age (LAZ)^c^, mean (SD)	−0.92 (0.99)
Early-infant weight-for-length (WLZ)^c^, mean (SD)	−0.55 (0.97)
Infant LAZ at 12 months, mean (SD)	−0.87 (0.97)
Infant WLZ at 12 months, mean (SD)	−0.56 (1.0)

^a^Household asset index was derived for the full MDIG cohort, and therefore quintiles are not each 20%.

^b^Category is primarily made up of those who partially fed human milk. Only one infant during the first 3 months and three infants during the first 6 month postpartum primarily did not feed human milk; however, there were weeks during those times when these infants were fed human milk, so they have been categorized with infants who were partially fed human milk.

^c^Early-infant measurement was each infant’s earliest measurement within the first 45 days of life (LAZ: *n* = 178; WLZ: *n* = 172).

In multivariable attained-size models, two individual HMOs (6’-sialyllactose (6’SL) and LnNT) were significantly inversely associated with 12-month LAZ after correction for multiple testing (Table 2). In the baseline-adjusted models, the associations between 6’SL and LNnT and LAZ were slightly attenuated but remained significant (Table 3). Both of these associations retained similar magnitudes and remained significant among infants who were primarily exclusively/predominantly fed human milk during the first 3 (or 6) months (Table 3). Conversely, the association for LnNT was attenuated in the relatively small group of infants categorized as primarily partially/not fed human milk in the first 3 or 6 months (Table 3). For 6’SL, the association with LAZ at 12 months was evident among infants classified as primarily partially/not fed human milk in the first 3 months, but the association was attenuated and non-significant among infants classified as primarily partially/not fed human milk in the first 6 months (Table 3). There was a weak correlation between 6’SL and LNnT (Pearson correlation coefficient (*r*) = 0.29) and effect estimates for both HMOs were slightly attenuated when the two HMOs were included in the same baseline-adjusted model for LAZ at 12 months (Table 3).

**Table 2:** Associations of length-for-age *z*-scores (LAZ) at 12 months of age with maternal human milk oligosaccharide (HMO) concentrations in a mother–infant cohort in Dhaka, Bangladesh.

HMO or HMO group	Unadjusted attained-size models (*n* = 192)		Adjusted attained-size models^a^ (*n* = 192)	
	Difference in mean LAZ^b^ (95% CI)	*p* value	Difference in mean LAZ^b^ (95% CI)	*p* value
Fucosylated or sialylated lactose HMOs^c^	0.0014 (−0.0013, 0.0041)	0.30	−0.0012 (−0.0070, 0.0047)	0.69
2’-Fucosyllactose (2’FL)	0.0015 (−0.0015, 0.0046)	0.32	−0.0011 (−0.0069, 0.0047)	0.70
3-Fucosyllactose (3FL)	0.016 (−0.0054, 0.038)	0.14	0.0079 (−0.014, 0.030)	0.48
3’-Sialyllactose (3’SL)	0.0027 (−0.021, 0.026)	0.82	0.0028 (−0.021, 0.026)	0.82
6’-Sialyllactose (6’SL)	−0.094 (−0.15, −0.037)	0.001	−0.090 (−0.14, −0.036)	0.001*
Difucosyllactose (DFLac)	0.017 (−0.014, 0.047)	0.28	0.012 (−0.030, 0.054)	0.57
Fucosylated, non-sialylated HMOs^c^	0.018 (0.0032, 0.033)	0.017	0.017 (0.0027, 0.032)	0.020*
Difucosyllacto-*N*-hexaose (DFLNH)	0.22 (−0.030, 0.47)	0.084	0.14 (−0.11, 0.40)	0.27
Difucosyllacto-*N*-tetrose (DFLNT)	0.013 (−0.0054, 0.032)	0.16	0.013 (−0.0085, 0.034)	0.24
Fucosyllacto-*N*-hexaose (FLNH)	0.080 (−0.26, 0.42)	0.64	0.16 (−0.19, 0.50)	0.38
Lacto-*N*-fucopentaose (LNFP) I	0.015 (−0.00072, 0.03)	0.062	0.0084 (−0.010, 0.027)	0.38
LNFP II	−0.0035 (−0.017, 0.0096)	0.60	0.0088 (−0.010, 0.028)	0.36
LNFP III	−0.21 (−0.53, 0.10)	0.18	−0.013 (−0.37, 0.35)	0.94
Non-fucosylated, sialylated HMOs^c^	−0.045 (−0.11, 0.024)	0.20	−0.015 (−0.085, 0.054)	0.67
Disialyllacto-*N*-hexaose (DSLNH)	−0.27 (−0.54, −0.0053)	0.046	−0.14 (−0.41, 0.13)	0.32
Disialyllacto-*N-*tetraose (DSLNT)	−0.013 (−0.10, 0.079)	0.78	−0.0017 (−0.090, 0.087)	0.97
Sialyl-lacto-*N*-tetraose b (LSTb)	−0.19 (−0.42, 0.034)	0.096	−0.084 (−0.37, 0.20)	0.57
Sialyl-lacto-*N*-tetraose c (LSTc)	−0.022 (−0.26, 0.22)	0.86	−0.0039 (−0.26, 0.25)	0.98
Fucosylated, sialylated HMOs (only includes fucodisialyllacto-*N*-hexaose (FDSLNH) in the group)^c^	−0.028 (−0.082, 0.026)	0.31	0.0010 (−0.067, 0.068)	0.98
Non-fucosylated, non-sialylated HMOs^c^	−0.019 (−0.033, −0.0053)	0.007	−0.018 (−0.032, −0.0046)	0.009*
Lacto-*N*-hexaose (LNH)	−0.027 (−0.36, 0.31)	0.87	−0.016 (−0.34, 0.31)	0.92
Lacto-*N*-neotetraose (LNnT)	−0.048 (−0.082, −0.013)	0.007	−0.047 (−0.081, −0.014)	0.006*
Lacto-*N*-tetrose (LNT)	−0.024 (−0.045, −0.0042)	0.018	−0.023 (−0.043, −0.0022)	0.031
Secretors (versus non-secretors)	0.20 (−0.091, 0.49)	0.18	0.059 (−0.23, 0.35)	0.69
Total HMO	0.0016 (−0.0016, 0.0047)	0.33	−0.0042 (−0.013, 0.0048)	0.36

*Significant following correction for multiple testing using Bonferroni–Holm method (target alpha used in calculation was <0.10).

^a^Adjusted for maternal BMI, maternal secretor status, mode of delivery, gravidity, maternal education, household asset index, maternal age, gestational age at birth, infant sex, vitamin D supplementation group, and infant feeding pattern (binary variable).

^b^Difference in mean LAZ per 100 nmol/ml increase in HMO concentration or HMO group concentration, or by secretor status.

^c^Fucosylated or sialylated lactose group = 2’FL + 3FL + DFLac + 3’SL + 6’SL; non-fucosylated, non-sialylated group = LNT + LNnT + LNH; fucosylated, non-sialylated group = LNFP I + LNFP II + LNFP III + DFLNT + FLNH + DFLNH; non-fucosylated, sialylated group = LSTb + LSTc + DSLNT + DSLNH; fucosylated, sialylated group = FDSLNH.

**Table 3:** Associations of length-for-age *z*-scores (LAZ) at 12 months of age with HMO concentrations adjusting for early-infant LAZ, overall and by infant feeding pattern.

HMO or HMO group	Baseline-adjusted models^a^ among all infants (*n* = 178)		Baseline-adjusted models^b^ among infants who primarily exclusively/predominately fed with human milk during first 3 months (*n* = 155)		Baseline-adjusted models^b^ among infants who primarily partially/not fed with human milk during first 3 months (*n* = 23)		Baseline-adjusted models^b^ among infants who primarily exclusively/predominately fed with human milk during first 6 months (*n* = 137)		Baseline-adjusted models^b^ among infants who primarily partially/not fed with human milk during first 6 months (*n* = 41)	
	Difference in mean LAZ^c^ (95% CI)	*p* value	Difference in mean LAZ^c^ (95% CI)	*p* value	Difference in mean LAZ^c^ (95% CI)	*p* value	Difference in mean LAZ^c^ (95% CI)	*p* value	Difference in mean LAZ^c^ (95%CI)	*p* value
HMO										
6’SL	−0.061 (−0.11, −0.012)^d^	0.015*	−0.058 (−0.11, −0.0043)	0.034	−0.27 (−0.51, −0.038)	0.028	−0.067 (−0.13, −0.0073)	0.028	−0.041 (−0.13, 0.045)	0.34
LNnT	−0.039 (−0.068, −0.010)^d^	0.008*	−0.038 (−0.068, −0.0073)	0.016	−0.023 (−0.15, 0.11)	0.69	−0.046 (−0.081, −0.011)	0.010	−0.012 (−0.064, 0.038)	0.61
6’SL (model also included LNnT)	−0.046 (−0.097, 0.0038)	0.070	–	–	–	–	–	–	–	–
LNnT (model also included 6’SL)	−0.032 (−0.061, −0.0018)	0.038	–	–	–	–	–	–	–	
HMO groups										
Fucosylated, non-sialylated HMOs^e^	0.016 (0.0035, 0.029)^d^	0.013*	0.017 (0.0029, 0.030)	0.018	0.020 (−0.055, 0.095)	0.56	0.022 (0.0062, 0.037)	0.006	−0.0073 (−0.030, 0.016)	0.52
Non-fucosylated, non-sialylated HMOs^e^	−0.013 (−0.025, −0.0014)^d^	0.029*	−0.013 (−0.026, 0.000057)	0.051	−0.012 (−0.060, 0.037)	0.60	−0.014 (−0.028, 0.00060)	0.060	−0.0091 (−0.029, 0.011)	0.36
Fucosylated, non-sialylated HMOs^e^ (model also included non-fucosylated, non-sialylated HMOs)	0.015 (0.0020, 0.027)	0.023	–	–	–	–	–	–	–	–
Non-fucosylated, non-sialylated HMOs^e^ (model also includede fucosylated, non-sialylated HMOs)	−0.012 (−0.024, 0.00018)	0.053	–	–	–	–	–	–	–	–

Note: cells in the table are blank because restricted analyses by infant feeding pattern were only conducted for the primary analyses with one individual or one group of HMOs as the primary exposure of interest.

*Bonferroni–Holm method used and remains significant with the correction for multiple testing (target alpha used in calculation was <0.05). To note, Bonferroni–Holm method for multiple testing was only used on models with only one individual or one group of HMOs among all infants as the exposure of interest.

^a^Adjusted for maternal BMI, maternal secretor status, mode of delivery, gravidity, maternal education, household asset index, maternal age, gestational age, infant sex, vitamin D supplementation group, infant feeding pattern (binary variable), and early-infant LAZ.

^b^Adjusted for maternal BMI, maternal secretor status, mode of delivery, gravidity, maternal education, household asset index, maternal age, gestational age, infant sex, vitamin D supplementation group, and early-infant LAZ.

^c^Difference in mean LAZ per 100 nmol/ml increase in HMO concentration or HMO group concentration.

^d^Individual and groups of HMOs were rescaled and standardized to their respective interquartile range (IQR) and produced the following difference in mean LAZ (95% CI) estimates: 6’SL: −0.17 (−0.31, −0.034), LNnT: −0.15 (−0.26. −0.038), fucosylated, non-sialylated HMOs: 0.16 (0.035, 0.29), non-fucosylated, non-sialylated HMOs: −0.13 (−0.24, −0.014).

^e^Fucosylated, non-sialylated group = LNFP I + LNFP II + LNFP III + DFLNT + FLNH + DFLNH; non-fucosylated, non-sialylated group = LNT + LNnT + LNH.

In the multivariable attained-size models, two HMO groups (non-fucosylated and non-sialylated HMOs; fucosylated and non-sialylated HMOs) had statistically significant associations with LAZ at 12 months after correcting for multiple testing (Table 2), non-fucosylated and non-sialylated HMOs were inversely associated with LAZ and fucosylated and non-sialylated HMOs were positively associated with LAZ. These associations were of similar magnitude and remained statistically significant in the corresponding baseline-adjusted models (Table 3). Both associations were of similar magnitude among infants who were primarily exclusively/ predominantly fed human milk during the first 3 or 6 months and among infants who were primarily partially/ not fed human milk during the first 3 months but were substantially attenuated among infants categorized as primarily partially/not fed human milk during the first 6 months (Table 3). There was no significant correlation between non-fucosylated, non-sialylated HMOs and fucosylated, non-sialylated HMOs (*r* = −0.16) and the effect estimates for both groups did not change when included in the same baseline-adjusted model for LAZ at 12 months (Table 3). There was no association between total HMO concentration and LAZ at 12 months in adjusted attained-size analyses (Table 2). Mean LAZ at 12 months postnatal age was higher in infants born to secretors (−0.81 (SD: 0.91)) versus non-secretors (−1.0 (SD: 1.1)) but the difference was not statistically significant (Table 2).

None of the individual HMOs, structurally similar HMO groups, total HMO concentration or secretor status were significantly associated with WLZ at 12 months in multivariable models after correction for multiple testing (Table 4).

**Table 4:** Associations of weight-for-length *z*-scores (WLZ) at 12 months of age with maternal human milk oligosaccharide (HMO) concentrations in a mother–infant cohort in Dhaka, Bangladesh.

HMO or HMO group	Unadjusted attained-size models (*n* = 192)		Adjusted attained-size models^a^ (*n* = 192)	
	Difference in mean WLZ^b^ (95% CI)	*p* value	Difference in mean WLZ^b^ (95% CI)	*p* value^c^
Fucosylated or sialylated lactose HMOs^d^	−0.0013 (−0.0041, 0.0015)	0.35	−0.0041 (−0.010, 0.0023)	0.21
2’FL	−0.0016 (−0.0047, 0.0016)	0.33	−0.0044 (−0.011, 0.0019)	0.17
3FL	−0.0025 (−0.025, 0.020)	0.83	0.00079 (−0.023, 0.025)	0.95
3’SL	0.0065 (−0.018, 0.031)	0.60	0.013 (−0.013, 0.038)	0.32
6’SL	−0.018 (−0.077, 0.041)	0.55	−0.018 (−0.078, 0.042)	0.55
DFLac	−0.013 (−0.045, 0.018)	0.41	−0.014 (−0.060, 0.031)	0.53
Fucosylated, non-sialylated HMOs^d^	0.0091 (−0.0065, 0.025)	0.25	0.011 (−0.0053, 0.026)	0.19
DFLNH	0.095 (−0.16, 0.35)	0.47	0.10 (−0.17, 0.38)	0.46
DFLNT	0.00017 (−0.019, 0.020)	0.99	0.0035 (−0.019, 0.027)	0.76
FLNH	0.26 (−0.083, 0.61)	0.14	0.37 (0.0035, 0.75)	0.048
LNFP I	0.00076 (−0.015, 0.017)	0.93	0.00085 (−0.019, 0.021)	0.93
LNFP II	0.0049 (−0.0085, 0.018)	0.47	0.011 (−0.0096, 0.032)	0.29
LNFP III	0.33 (0.0099, 0.66)	0.043	0.50 (0.11, 0.88)	0.012
Non-fucosylated, sialylated HMOs^d^	0.0099 (−0.062, 0.081)	0.79	0.022 (−0.053, 0.098)	0.56
DSLNH	−0.038 (−0.31, 0.24)	0.79	−0.0061 (−0.30, 0.29)	0.97
DSLNT	0.021 (−0.073, 0.11)	0.67	0.028 (−0.068, 0.12)	0.56
LSTb	0.031 (−0.2, 0.27)	0.79	0.068 (−0.24, 0.38)	0.67
LSTc	−0.029 (−0.28, 0.22)	0.82	0.019 (−0.26, 0.30)	0.89
Fucosylated, sialylated HMOs (only includes FDSLNH in the group)^d^	0.048 (−0.0077, 0.10)	0.091	0.068 (−0.0037, 0.14)	0.063
Non-fucosylated, non-sialylated HMOs^d^	0.00070 (−0.014, 0.015)	0.93	−0.0014 (−0.017, 0.014)	0.86
LNH	0.18 (−0.17, 0.52)	0.31	0.19 (−0.17, 0.54)	0.30
LNnT	−0.0034 (−0.040, 0.033)	0.85	−0.0091 (−0.046, 0.028)	0.63
LNT	0.0020 (−0.019, 0.023)	0.86	−0.00037 (−0.023, 0.022)	0.98
Secretor status	−0.0440 (−0.35, 0.26)	0.77	−0.14 (−0.45, 0.17)	0.38
Total HMO	−0.0012 (−0.0044, 0.0021)	0.48	−0.0046 (−0.014, 0.0052)	0.36

^a^Adjusted for maternal BMI, maternal secretor status, mode of delivery, gravidity, maternal education, household asset index, maternal age, gestational age, infant sex, vitamin D supplementation group, and infant feeding pattern (binary variable).

^b^Difference in mean WLZ per 100 nmol/ml increase in HMO concentration or HMO group concentration, or by secretor status.

^c^None of the associations remained significant with the correction for multiple testing using Bonferroni–Holm method (target alpha 0.10 used in calculation to determine significance level).

^d^Fucosylated or sialylated lactose group = 2’FL + 3FL + DFLac + 3’SL + 6’SL; non-fucosylated, non-sialylated group = LNT + LNnT + LNH; fucosylated, non-sialylated group = LNFP I + LNFP II + LNFP III + DFLNT + FLNH + DFLNH; non-fucosylated, sialylated group = LSTb + LSTc + DSLNT + DSLNH; fucosylated, sialylated group = FDSLNH.

In sensitivity analyses conducted using four alternative HMO groups based on specific chemical linkages, HMOs with alpha 2-6-linked sialic acid to the terminal galactose (a group comprised of 6’SL and LSTc) was associated with lower LAZ at 12 months (Supplementary Table 2). None of the four groups based on linkages were significantly associated with WLZ at 12 months (Supplementary Table 3). Inferences from models of LAZ at 12 months regressed on individual HMOs, structurally similar groups of HMOs, total HMO concentration, and secretor status were similar to the primary analysis when adjusting for the number of weeks exclusive/predominantly fed human milk during the first 3 months postnatally, instead of the categorical infant feeding pattern variable (data not shown). Inferences from the baseline-adjusted models were also the same as the primary analysis when using alternative age cut-offs for the baseline LAZ measurement: within 2 days of life (*n* = 138) and the earliest LAZ measured for each participant that included up to 100 days postpartum (*n* = 192) (data not shown). Estimates from the baseline-adjusted models restricted to term infants were similar to those including all infants but were less precise, although generally supportive of the primary inferences (Supplementary Table 4).

## Discussion

In a mother–infant cohort in Dhaka, Bangladesh, infant length at 12 months was associated with specific individual and structurally similar groups of HMOs. These findings add to the emerging body of evidence that specific HMOs influence infant growth^27–35^ by considering the association of structurally similar HMOs with infant length and investigating inferences based on infant feeding patterns.

In the present study, LNnT and the group of non-fucosylated non-sialylated HMOs (which includes LNnT) were associated with reduced LAZ at 12 months. It is likely that the inverse association for the group of non-fucosylated non-sialylated HMOs was primarily driven by the effect of LNnT. Considering a plausible change in an infant’s LNnT exposure from the 25th to 75th percentile (437–816 nmol/ml),^48^ the 0.16 lower LAZ expected from this approximate 400 nmol/ml increase in LnNT would be substantial in a context in which mean LAZ is already compromised (average of −0.9). The present study design does not enable conclusions about whether this effect is causal; however, these findings are consistent with several other studies that similarly found LNnT to be inversely related with infant linear growth at a variety of infant ages and geographical locations.^29,30,32,33,35^ In a study conducted among mother–infant pairs pooled from seven European countries (France, Italy, Norway, Portugal, Romania, Spain, and Sweden), there was a significant negative correlation between LNnT and change in length from birth to four months.^35^ The negative association between LNnT and LAZ has also been reported in a study with long-term follow-up for infants aged 3–12 months and 1–5 years in Finland, in which HMOs were measured in human milk at three months.^30^ However, a small (*n* = 175) randomized controlled trial of an infant formula with added LNnT and 2’FL given for 6 months postpartum did not find significant effects on infant anthropometric outcomes over the first year of life compared to infants given the same formula without LNnT and 2’FL.^36^ The concentration of LNnT in the trial formula (0.5 g/l) was similar to the median concentration of naturally occurring LNnT (0.43 g/l (IQR: 0.31, 0.58 g/l)) in the present study population,^48^ but perhaps the sample size was too small to see a between-group difference in anthropometric outcomes. Furthermore, a trial of a synbiotic (*B. infantis* probiotic + LNnT), probiotic alone, compared to placebo conducted among Bangladeshi infants 2–6 months old with severe acute malnutrition (SAM) did not report a significant effect of the intervention on infant linear growth even though the amount of LNnT given (1.6 g approximately 2–3 times daily depending on the volume of feed required for the baby while in hospital and 1.6 g twice daily upon discharge)^61^ was higher than the naturally occurring concentration. The population of this synbiotic trial was different in terms of linear growth compared to the present study population: LAZ was much lower at enrolment (mean (SD): −2.0 (1.3) in trial versus −0.92 (0.99) in the present study) and at final follow-up (mean (SD): −1.7 (1.2) in trial versus −0.87 (0.97) in this study). Following from these two randomized controlled trials, it is possible that other probiotics or prebiotics (i.e., 2’FL) modify the effect of LNnT on growth.

The individual HMO 6’SL was also associated with lower LAZ at 12 months in the present study and the magnitude of effect on infant linear growth was larger than compared to LNnT per 100 nmol/ml increase. However, the concentration range was narrower for 6’SL compared to LNnT, and the magnitudes of effects on infant linear growth were similar when the concentrations were alternatively scaled according to each HMO’s respective IQR. It is likely that the inverse association for the chemically similar group of alpha 2-6-linked sialic acid to terminal galactose (6’SL + sialyl-lacto-*N*-tetraose c (LSTc)) was primarily driven by the effect of 6’SL; estimates for LSTc were closer to the null yet it was also inversely associated with LAZ at 12 months. Other observational studies have not reported an association between 6’SL and infant linear growth; however, two in vivo studies using mouse and piglet models fed with a purified sialylated bovine milk oligosaccharide (S-BMO) mixture made up mainly of 3’SL and 6’SL, but also contains other sialylated HMOs that are not present in human milk, found that supplementation promoted microbiota-dependent linear growth among animals colonized with feces from growth-stunted infants from Malawi and Bangladesh.^25,26^ There have been reports of negative associations between 6’SL and other measures of infant body composition such as weight, fat mass,^32^ body mass index,^29^ and head circumference^27^ in the literature. A small number (*n* = 45) of infants from the synbiotic (*B. infantis* + LNnT) trial conducted in Dhaka, Bangladesh previously described were included in a sub-study that found that a higher relative abundance of sialylated HMOs (which included but was not limited to 6’SL) was associated with an increased odds of SAM, defined as WLZ < −3, compared to non-malnourished infants.^62^ In that study, 58% of the infants had SAM with a mean WLZ of −3.8 ± 0.33, so the larger magnitude of association may have been due to differences in the baseline nutritional status of the two cohorts.

There was a weak correlation between 6’SL and LNnT, which resulted in a slight attenuation of effects seen in models including both HMOs. Pell et al. has previously published correlations between individual HMOs among this study population and reported that the correlation between 6’SL and LNnT varied by secretor status.^48^ Secretor status acts as a global indicator for variations in HMO concentrations and as such the individual concentration of 6’SL and LNnT and their subsequent impact on infant linear growth may be influenced by secretor status. To account for this we included secretor status as a covariate in adjusted models in order to make the results as generalizable to the public as possible, whereas some other observational studies chose to stratify analyses by secretor status^27,30^ or assess the impact with an interaction term between secretor status and individual HMOs.^32,33^

As a group, fucosylated, non-sialylated HMOs (including difucosyllacto-*N*-hexaose (DFLNH), difucosyllacto-*N*-tetrose (DFLNT), fucosyllacto-*N*-hexaose (FLNH), lacto-*N*-fucopentaose (LNFP) I, LNFP II, and LNFP III) were associated with higher LAZ at 12 months. Among this group, DFLNH has one of the strongest positive effects with LAZ (although not significant), which has been reported in other observational mother–infant cohorts from Gambia at 5 months postpartum^28^ and from Australia at three months postpartum.^32^ Among the cohort of mother–infant dyads from Gambia, Davis et al. also reported a significant positive association between LNFP I and III and LAZ at 5 months.^28^ Davis et al. also investigated associations between HMOs and infant gut microbiota composition and morbidity and suggested that fucosylated HMOs, which included but was not limited to the fucosylated, non-sialylated HMOs in the present study, may promote infant growth through microbiota-mediated pathways and indirectly through prevention of infection.

A strength of this study was that the cohort consisted of a majority of infants who were primarily exclusively/predominately fed human milk but also included infants who were primarily partially/did not feed with human milk for the majority of the time during the first 3 and 6 months postpartum, so that we could investigate a dose-response phenomenon, assuming that exclusively/predominately human milk fed infants received the highest quantities of HMOs. This is unlike other observational studies of associations between HMOs and infant health outcomes that only included infants who were exclusively fed human milk or only consider infant feeding patterns as a confounder in adjusted models, which does not address the variability in infant feeding patterns during the postnatal period that affect volume of human milk consumed and therefore the extent of exposure to HMOs. A few observational studies have used intake of individual HMOs measured by 24-hour test weighing and infant body composition as the exposure of interest in models with infant body composition outcomes^29,32,63^ and these types of approaches may help clarify the association between HMOs and growth in future studies.

This study was limited by a small sample size, such that meaningful associations may not have been estimated with sufficient precision. In addition, infant feeding pattern was collected longitudinally in the present study, with data missing from nearly 10% of visits, and the number of infants who primarily partially/ did not feed with human milk during the first 3 and 6 months was low overall; therefore, inferences regarding the modifying effects of infant feeding pattern need to be interpreted with caution. Comparisons between studies of human milk components are challenged by variations in methods of milk sample collection, including number and timing of samples, and HMO analysis. The present study was limited by only having one human milk sample per participant, since HMO concentrations change during lactation.^17^ In addition, like most studies of HMOs and infant health outcomes this study was observational and therefore cannot account for all confounding factors that affect both human milk feeding initiation or duration and child growth. Therefore, randomized trials specifically designed to measure the effect of HMOs on infant growth and other health outcomes are needed.

It has been well documented that HMOs influence the infant gut microbiome^64–66^; however, more research is needed on the interaction between specific HMOs or HMO profiles and microbiome profiles,^1^ and their combined effects on health outcomes. The present findings contribute to the growing evidence of the relationships between human milk components and infant growth and have implications for the design of interventions that may modulate HMO consumption by infants to support healthy childhood growth and development.

### Supplementary Information

Supplementary Table 1

Supplementary Tables 2, 3, 4

## Data Availability

Data described in the manuscript, code book, and analytic code will be made available upon request to the authors. De-identified individual participant data will be provided for use in secondary data analyses approved by an independent research ethics board, and data requestors will be required to sign a data access agreement.
