# Supplementary material for: Associations between human milk oligosaccharides and infant growth in a Bangladeshi mother–infant cohort
Source: Pediatr Res. 2023 Dec 5;96(2):356–64. doi: 10.1038/s41390-023-02927-1 (PMC11343707; doi:10.1038/s41390-023-02927-1)
Supplement: Supplementary file 2 — Supplementary Tables 2, 3, 4 [file 41390_2023_2927_MOESM2_ESM.pdf]

Supplementary Table 2. Associations of length-for-age z-scores (LAZ) at 12 months of age with maternal HMO groups based on chemical linkages

**HMO group based on chemical linkages**	**Unadjusted attained-size models (n=192)**		**Adjusted attained-size models (n=192)[a]**		**Baseline-adjusted models (n=178)[b]**	
	**Difference in mean LAZ[c] (95%CI)**	**p-value**	**Difference in mean LAZ[c] (95%CI)**	**p-value**	**Difference in mean LAZ[c] (95%CI)**	**p-value**
alpha 1-2-linked fucose (LNFP I + 2'FL)	0.0017 (-0.0010, 0.0044)	0.23	-0.00025 (-0.0054, 0.0049)	0.92	-	-
alpha 2-6-linked sialic acid to terminal galactose (6SL + LSTc)	-0.090 (-0.14, -0.035)	0.001	-0.086 (-0.14, -0.033)	0.002	-0.058 (-0.11, -0.010)	0.017
alpha 2-3-linked sialic acid to terminal galactose (3SL+ DSLNT)	0.0017 (-0.021, 0.024)	0.88	0.0024 (-0.020, 0.025)	0.83	-	-
alpha 2-6-linked sialic acid to internal N-acetylglucosamine (LSTb +DSLNT)	-0.027 (-0.099, 0.045)	0.46	-0.0063 (-0.078, 0.066)	0.86	-	-

[a]Adjusted for maternal BMI, maternal secretor status, mode of delivery, gravidity, maternal education, household asset index, maternal age, gestational age, infant sex, vitamin D supplementation group, and primary infant feeding pattern during first 3 months of life (binary variable).
[b]Adjusted for maternal BMI, maternal secretor status, mode of delivery, gravidity, maternal education, household asset index, maternal age, gestational age, infant sex, vitamin D supplementation group, primary infant feeding pattern during first 3 months of life (binary variable), and early-infant LAZ
[c] Difference in mean LAZ per 100 nmol/ml increase in HMO group concentration

Supplementary Table 3. Associations of weight-for-length z-scores (WLZ) at 12 months of age with maternal HMO groups based on chemical linkages

HMO group based on chemical linkages	Unadjusted attained-size models (n=192)		Adjusted attained-size models (n=192)[a]	
	Difference in mean WLZ[b] (95%CI)	p-value	Difference in mean WLZ[b] (95%CI)	p-value
alpha 1-2 fucose (LNFP I + 2'FL)	-0.0012 (-0.0040, 0.0016)	0.40	-0.0033 (-0.0089, 0.0022)	0.24
alpha2-6 sialic acid to terminal gal (6SL + LSTc)	-0.019 (-0.077, 0.039)	0.53	-0.016 (-0.075, 0.042)	0.58
a2-3-linked sialic acid to terminal Gal (3SL+ DSLNT)	0.0071 (-0.016, 0.030)	0.55	0.013 (-0.011, 0.037)	0.28
a2-6-linked sialic acid to internal GlcNAc (LSTb +DSLNT)	0.016 (-0.058, 0.090)	0.67	0.023 (-0.055, 0.10)	0.56

[a]Adjusted for maternal BMI, maternal secretor status, mode of delivery, gravidity, maternal education, household asset index, maternal age, gestational age, infant sex, vitamin D supplementation group, and primary infant feeding pattern during first 3 months of life (binary variable).

[b] Difference in mean WLZ per 100 nmol/ml increase in HMO group concentration

Supplementary Table 4. Associations of length-for-age z-scores (LAZ) at 12 months of age with HMO concentrations adjusting for early-infant LAZ among term infants for individual HMOs and groups of HMOs significant in baseline-adjusted models among all infants (estimates from baseline-adjusted models among all infants from Table 3 are repeated here for ease of comparison between models).

HMO or HMO group	Baseline-adjusted models[a] among all infants (n=178)		Baseline-adjusted models[a] among term infants (n=166)	
	Difference in mean LAZ[c] (95%CI)	p-value	Difference in mean LAZ[c] (95%CI)	p-value
**HMO**				
6'SL	-0.061 (-0.11, -0.012)	0.015	-0.046 (-0.098, 0.0063)	0.084
LNnT	-0.039 (-0.068, -0.010)	0.008	-0.036 (-0.065, -0.0064)	0.017
**HMO Groups**				
Fucosylated, non-sialylated HMOs[b]	0.016 (0.0035, 0.029)	0.013	0.016 (0.0027, 0.029)	0.019
Non-fucosylated, non-sialylated HMOs[b]	-0.013 (-0.025, -0.0014)	0.029	-0.012 (-0.024, 0.00037)	0.057

[a]Adjusted for maternal BMI, maternal secretor status, mode of delivery, gravidity, maternal education, household asset index, maternal age, gestational age, infant sex, vitamin D supplementation group, infant feeding pattern (binary variable), and early-infant LAZ

[b]Fucosylated, non-sialylated group = LNFP I + LNFP II + LNFP III + DFLNT + FLNH + DFLNH; Non-fucosylated, non-sialylated group = LNT + LNnT + LNH

[c] Difference in mean LAZ per 100 nmol/ml increase in HMO concentration or HMO group concentration
